# Sperm handling and management in the teleost model fish Japanese medaka (*Oryzias latipes*)

**DOI:** 10.1038/s41598-024-65376-8

**Published:** 2024-06-26

**Authors:** Amin Sayyari, Anette Kristine Krogenæs, Ian Mayer, Catherine Labbé

**Affiliations:** 1 Department of Production Animal Clinical Sciences, Faculty of Veterinary Medicine, Norwegian University of Life Sciences, Ås, Norway; 2 INRAE, Fish Physiology and Genomics, UR 1037, Rennes, France

**Keywords:** Japanese medaka, Sperm collection, Sperm motility, Short-term storage, Cryopreservation, Reproductive biology, Animal biotechnology

## Abstract

Japanese medaka (*Oryzias latipes*) has been used as a model organism in different research fields, including reproductive physiology. Sperm motility is the most important marker for male fertility in fish and, thus, reproduction success. However, because of small volume of ejaculate and short motility duration, it is still challenging to manage the sperm collection and analysis in small model fish. In the present study, we aimed to investigate sperm motility and to optimize sperm collection, short-term sperm storage, and cryopreservation in Japanese medaka (*Oryzias latipes*). Using two different approaches for sperm collection: testes dissection and abdominal massage, different housing conditions and activating the sperm with different activation solutions, we investigated immediate sperm motility. In the second part of this study, we used different osmolalities of immobilization solution, Hank's Balanced Salt Solution (HBSS) for sperm storage at 0, 2 and 3 h after sperm collection. Finally, the sperm were cryopreserved using methanol as cryoprotectant and HBSS as extender at two different osmolalities, and post-thaw sperm motility was investigated. The highest post-activating sperm motility was achieved in the groups activated by the extender at 300 mOsm/kg. The quality of sperm remained unaffected by co-housing with females or with males only. Furthermore, Hanks’ Balanced Salt Solution (HBSS) with an osmolality of 600 mOsm/kg demonstrated its efficacy as a suitable extender for sperm storage, preserving motility and progressivity for 3 h. The highest post-thaw motility was around 35%. There were no significant differences between post-thaw motility in different groups. We also found that post-thaw incubation on ice can maintain the motility of the sperm for up to one hour after thawing.

## Introduction

Medaka (*Oryzias latipes*) is an attractive model organism for the study of fish reproduction. Medaka is used for the evaluation of the spawning cycle as well as the transgenerational studies because the spawning is easily induced, and maturation time is relatively short^1,2^. These advantages have made it appropriate for using medaka as a model organism for functional genomics and epigenetics^3,4^, reproductive physiology^5,6^ and toxicology^7^. As medaka has become a more and more popular vertebrate model system in various research fields, methods for sperm handling and management have become necessary for experimental approaches. Recently, most researchers that used medaka sperm characteristics as endpoint extracted milt by crushing dissected testes^3,8^ and to the best of our knowledge, the two available published articles about medaka sperm handling and storage are based on sperm collection after testes dissection^2,9^. Extracting the milt directly into the activating medium by abdominal massage has also been used in some previous studies^10–12^, however, there are some limitations with this method as visualizing the amount and color of extracted milt are difficult, recording milt volume is impossible and dilution variations are unavoidable. Medaka sperm collection by abdominal massage has been demonstrated in a methodological study where a protocol for sperm analysis by CASA was described^13^. However, sperm handling and management in medaka as a model organism needs to be improved, as it provides an important basis for further research into in vitro fertilization, cryopreservation, and epigenetics.

Sperm motility is considered as the most important biomarker for male fish fertility and reproductive success^14^. Spermatozoa are maintained in most fish species at the rest stage and are immotile in the testis. The sperm motility will be triggered when sperm is released into an activation medium which is hypotonic in freshwater fish or hypertonic in marine fish^14^. Nevertheless, medaka sperm motility can be induced by hypertonic, isotonic, or hypotonic medium, depending on previous seawater or freshwater adaptation^2^. From sperm collection to evaluation, it is important to avoid motility activation due to the short motility longevity, and this is also true in most fish species. There are different solutions that can be used for fish sperm immobilization. These solutions mimic seminal plasma composition, osmolality, and pH^14^. The other factors that can influence fish sperm motility characteristics are temperature and concentration of metabolites and ions (CaCl_2_, MgCl_2_, etc.) in fish swimming environment^15^. Sperm collected using various methods are either analyzed immediately after collection or are stored in immobilization medium for later analysis. Optimizing sperm handling prior to short-term and long-term storage is essential to reduce sperm motility loss, especially when cryopreservation is intended. Factors such as proper sperm collection and storage conditions including the use of immobilization medium, the right temperature and pre-freezing storage duration time are quite decisive for having increased post-thaw motility. In addition, studies on proper sperm handling in small model fish will also provide useful information for other teleost fishes of aquaculture interest. Therefore, the goal of this present study is to investigate the handling procedures of medaka sperm, from collection to further purposes such as sperm evaluation and/or cryopreservation. We specifically evaluated various factors affecting the sperm motility characteristics in the handling process: fish housing conditions, sperm collection methods, sperm activation (osmolality), choice of extenders and storage duration.

## Materials and methods

### Ethics statement

The study was conducted in the Fish Physiology and Genomics department (LPGP) at INRAE (Rennes, France) fish facility. All fish were reared in the ISC INRAE LPGP fish facility, which hold full for experimental fish rearing in strict line with French and European Union Directive 2010/63/EU for animal experimentation (agreement number: D-35-238-6). The experiments and handling of fish were approved by the welfare committee of the Fish Physiology and Genomics department at INRAE (registration O-2022-01-CL) in agreement with the guidelines for care and use of laboratory animals and in compliance with French and European Union regulations on animal welfare (agreement n°005239, C. Labbé). All fish were handled for sperm collection in strict accordance with the guidelines of the Institutional Animal Care and Use Ethical Committee in Rennes LPGP (Fish Physiology and Genomics Department). CL is accreted by the French Veterinary Authority for fish experimentation (no. 005239). The animal study is reported in accordance with ARRIVE guidelines (https://arriveguidelines.org) for animal research.

### Sperm collection

Medaka fish (*Oryzias latipes*) were from the CAB strain. The fish used in this study were 6 to 9 months old with body lengths of 3.0 ± 0.4 cm (mean ± SD). They were reared in re-circulating water system at 26 °C and the photoperiod was set at 14 h light: 10 h dark. The fish were fed twice per day with Gemma micro 500 (Skretting, Norway) and once per day with baby brine shrimps (*Artemia* spp.). Methods for sperm collection are described in our previous methodological study^13^. We used the same methods with some adjustments where needed. Briefly, for stripping, we anesthetized the male medaka in TRIS buffered tricaine methane sulfonate solution MS-222 at 225 mg/L supplemented with NaHCO_3_ at 450 mg/L. After anesthesia, the fish were blotted with a paper towel to dry the body. Then, the fish was placed in the holding sponge under a dissecting microscope in dorsal recumbency (belly up). The calibrated micro-capillary tube with aspirator (Drummond Scientific, Pennsylvania, USA) was attached against the cloaca of the fish. Sperm was collected by gently squeezing the abdomen with blunt end smooth forceps in a rostral to caudal motion while simultaneously sucking to collect the expelled milt into the tubes. After measuring volume and macroscopic evaluation of the milt, the sample was transferred to 1.5-mL centrifuge tube containing medium for further analysis (see the next sections for more details). Based on the breeding and housing strategy of the fish facility, the fish used for sperm collection under anesthesia were either recovered or euthanized after the procedure. For testes dissection, male fish were euthanized by immersion in a lethal dose of MS-222 at 300 mg/L supplemented with NaHCO_3_ at 600 mg/L. The testes were removed and separated from surrounding lipid tissues while viewing with a dissecting microscope (× 10 magnification) and transferred to 1.5-mL centrifuge tube containing sperm activation medium for immediate sperm motility analysis.

### Sperm motility evaluation

Total motility was analyzed using computer assisted sperm analysis (CASA) and IVOS II system (IMV technologies) using Leja chamber slides (Leja, Netherlands) with a depth of 20 µm (Leja20, four-chamber, LOT: 481815B1) (see Supplementary Video S1). The settings for CASA software were adapted for medaka based on our previous study^13^. The following CASA parameters were analyzed: (1) total motility (%); (2) progressive motility (%); (3) kinematics parameters: curvilinear velocity (VCL, µm/s), straight-line velocity (VSL, µm/s), average path velocity (VAP, µm/s), linearity (LIN = VSL/VCL, %), wobble (WOB = VAP/VCL, %) and straightness (STR = VSL/VAP, %). For each sample, the sperm motility was estimated with at least three fields observed each time. Each field consisted in a 30 frames movie (camera speed 60 images/sec). Sperm were considered progressive if the straightness index (STR) was > 68% (i.e. sperm turning in circle are considered not progressive).

### Sperm motility initiation study

Before testing the sperm activation with different activation media, spontaneous sperm motility was evaluated in 8 males. Sperm were collected individually by abdominal massage in a 10 µL micro-capillary tube. A drop of milt was loaded onto a clean slide glass and sperm motility was estimated at × 200 magnification using dark-field microscopy (LEITZ DMRB, Leica). In this experiment, sperm motility parameters were evaluated using different activation solutions, collecting methods and different housing conditions.

#### Testing activation solutions

In our previous study, we found that medaka sperm were motile in HBSS (287 mOsm/kg)^13^, in accordance with the finding from Yang et al.^2^. Therefore, we chose this osmolality (~ 300 mOsm/kg hereafter as HBSS 300) for testing sperm activation in two different activation solutions (prepared in the laboratory by the authors): (1) Hanks’ balanced salt solution (HBSS 300) (137 mM NaCl, 5.4 mM KCl, 1.3 mM CaCl_2_, 1.0 mM MgSO_4_, 0.25 mM Na_2_HPO_4_, 0.44 mM KH_2_PO_4_, 4.2 mM NaHCO_3_, and 5.55 mM glucose, pH 7.2, 300 mOsm/kg) and (2) Kurokura (K 180) (180 mM NaCl, 2.68 mM KCl, 1.36 mM CaCl_2_, and 2.38 mM NaHCO_3_). The later, Kurokura (K 180), will be referred to as K 300 in this study with regards to its osmolality. Milt was individually collected from 19 individual males and all the samples were immediately mixed with their respective activation solutions (HBSS 300, n = 13 and K 300, n = 7). Sperm was activated at room temperature (RT) by quickly mixing the sample. To obtain the optimal test temperature, the tubes of activation solutions were placed on a water bath at 28 ± 1 °C. To obtain an optimal testing concentration, the dilution factor was chosen based on the volume and density of the milt. The 1.5-mL Eppendorf tubes containing 200–500 µL activation medium were prepared before sampling. The time between activation and analysis was registered and was under 10 s for almost all the samples.

#### Evaluation of sperm motility in samples collected by two methods: abdominal massage (stripping) versus testes dissection

Nineteen males medaka were used to evaluated sperm motility with two different collection methods. Milt was collected by abdominal massage and analyzed as described before. Shortly after analyzing, the testes from the same 19 males were dissected and the retrieved sperm suspension was analyzed immediately after mixing the dissected testes with activation medium. The time between sample activation and analysis was recorded and was under 10 s for both collection methods. The activation medium used in this experiment was HBSS 300.

#### Evaluation of sperm motility in two different housing conditions: males separated from females for around one month vs. males separated from females one night prior to collection

Milt was stripped from 7 separated males and 6 males housed with females. By mixing the milt with activation medium (HBSS 300), sperm motility parameters were analyzed under 10 s after milt collection.

### Sperm motility duration

To evaluate sperm motility duration, after being activated in HBSS 300, sperm suspensions from 6 individual males were stored at RT, and motility was evaluated at 0, 30, and 60 min after activation.

### Sperm storage study

#### Short-term storage

For practical reasons, it is necessary to store sperm samples in appropriate conditions. Choice of immobilization medium, storage temperature and storage time are among factors that can affect sperm viability and quality. This is especially important when it is necessary to store the milt before in vitro* fertilization* (IVF), cryopreservation, microscopic and molecular assessments.

Milt from 16 individual males (four males were later excluded because of poor initial sperm quality) was collected into 16 µL of HBSS with osmolality of 600 mOsm/kg (HBSS 600), and evenly divided into 3 tubes containing HBSS 0 (n = 12), HBSS 400 (n = 12) and HBSS 600 (n = 12) to yield a final osmolality of 300, 500 and 600 mOsm/kg, respectively. The motility and velocity were evaluated at 0, 2, and 4 h post collection (hpc). In this experiment, the sperm were activated immediately after preparation of sperm suspension in HBSS 300 as initial motility (0 hpc). At the same time (0 hpc), the milt from HBSS 500 and 600, were subjected to immobilization test. At 2 and 4 hpc, the sperm in HBSS 300, 500, 600 were used to activation evaluation and sperm in HBSS 500 and 600 were further used for immobilization test. For motility evaluation by CASA, 1 µL of sperm suspension in each tube was added into a tube containing 10 µL HBSS of adjusted osmolality to yield a final osmolality of 300 mOsm/kg. For immobilization test, 1 µL of sperm suspension from HBSS 500 and 600 was added into a tube containing 10 µL HBSS with corresponding osmolalities and the percentage of immobile spermatozoa was then recorded by CASA. The samples were placed on ice during short-term storage test study.

#### Long-term storage (cryopreservation)

The milt was individually collected from 10 males and added to storage medium in different immobilization solutions (HBSS and Kurokura) at the osmolality of around 600 mOsm/kg. Sperm motility of fresh milt samples was evaluated by CASA after around one hour storage on ice. The sperm cryopreservation method was described previously by Depincé et al.^16^. Briefly, the individual sperm samples were divided into different replicates and one volume of each replicate was mixed with one volume of cryopreservation solution consisting of experimental storage solutions at 600 mOsm/kg with 16% (vol) methanol and 126mM sucrose. Aliquots (60 μL) of sperm suspension were loaded into 250-μl French straw (IMV International, Minneapolis, MN, USA) by pipetting. The samples were cooled with a cooling rate of − 10 °C/min from + 2 °C to − 80 °C in a Planer Kryo 360 controlled-rate cooler (Planer plc, UK) and straws were then transferred into liquid nitrogen. Samples were evaluated at the first day of storage in liquid nitrogen. Each straw was thawed individually in a water bath at 40 °C for 5 s, wiped dry with a paper towel, and was released into a sterile 1.5-ml centrifuge tube by cutting of the end (with the cotton plug). The samples were analyzed by CASA after thawing and cryosurvival factor (CSF) was calculated by $CSF =  (\%\text{total motile sperm post}-\text{thaw})/(\%\text{total motile sperm fresh})\times 100$. After post-thaw analysis, the rest of the sample was placed on ice, and the samples were analyzed by CASA after one hour storage on ice as described before. For post-thaw storage trials, milt samples from 10 individual males were added to immobilization solutions at different osmolalities of HBSS and Kurokura. This yielded sixteen cryopreserved samples to analyze for post-thaw storage on ice (see Supplementary Fig. S1).

### Data analysis

The normality of distribution was controlled by residual and predicted values plot, normal-percentile plots, and Shapiro–Wilk test. If the *p*-value in the Shapiro–Wilk test was over 0.05, data were considered normally distributed. Data that were not normally distributed were transformed or analyzed by non-parametric tests, such as Mann–Whitney *U*. The effect of extenders, collection methods and housing conditions on motility, progressivity, VCL, and VSL was performed by independent samples *t*-test. One-way ANOVA analyses were conducted to find the influence of storage time on total sperm motility, progressivity, VCL, and VSL. The results are presented as mean ± S.E. The male-specific effects were handled by incorporating individual variances as random effects and nesting them within different experimental designs using a mixed model approach. All analyses were performed at a significant level of 0.05 using JMP®, Version 16 (SAS Institute Inc., Cary, NC, USA). All figures were generated using GraphPad Prism (9.0.0 for Windows, GraphPad Software, Boston, Massachusetts USA, www.graphpad.com).

## Results

### Sperm motility initiation study

#### Spontaneous motility of sperm

No spontaneous movement before activation of the sperm collected from 8 males was recorded.

#### Testing different activation solutions

**Figure 1:**
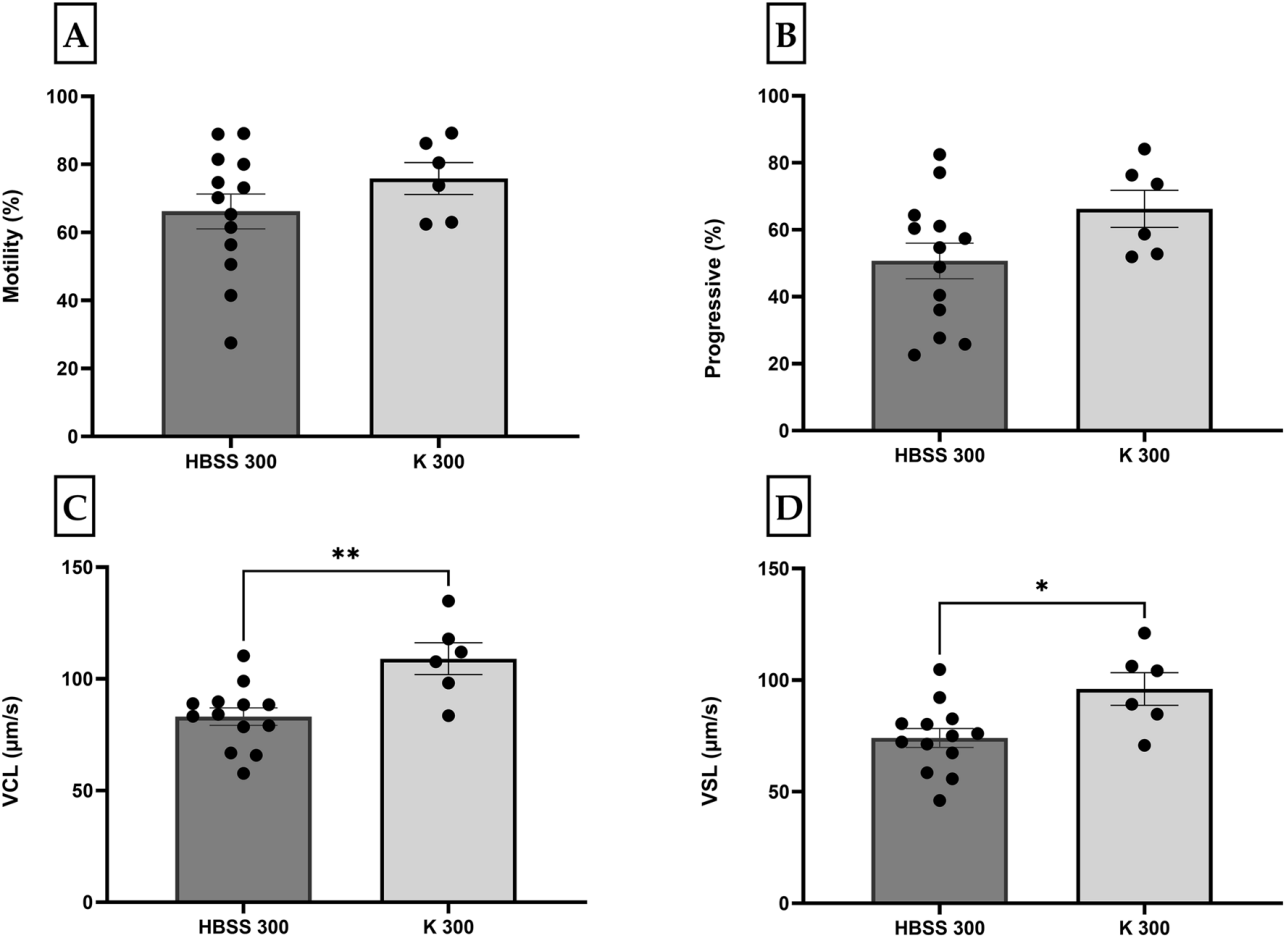
Motility parameters (Mean ± SE) of medaka spermatozoa immediately after milt collection activated by HBSS 300 (n = 13) and Kurokura (K 300) (n = 6): (**A**) motility rate (%); (**B**) progressivity (%); (**C**) curvilinear velocity (VCL) (μm/s); and (**D**) straight-line velocity (VSL) (μm/s). The figures present data sets that are significant at different levels: * p < 0.05, ** *p* < 0.01.

Sperm movement was tested immediately after activation in HBSS 300 and K 300. The total sperm motility was non-significantly higher in sperm activated by K 300 (75 ± 5%, *p* = 0.18) compared to HBSS 300 (66 ± 5%) (Fig. 1A). The same effect was recorded in progressivity in K 300 (66 ± 6%, *p* = 0.06) compared to HBSS 300 (51 ± 5%) (Fig. 1B). Significantly higher VCL (109 ± 7 µm/s, *p* = 0.009) and VSL (96 ± 7 µm/s, *P* = 0.03) were observed in sperm activated by K 300 compared to HBSS 300, VCL (83 ± 4 µm/s) and VSL (74 ± 4 µm/s) (Fig. 1C,D). Similar significant differences were observed in VAP (see Supplementary Table S1).

#### Testing different collection methods

**Figure 2:**
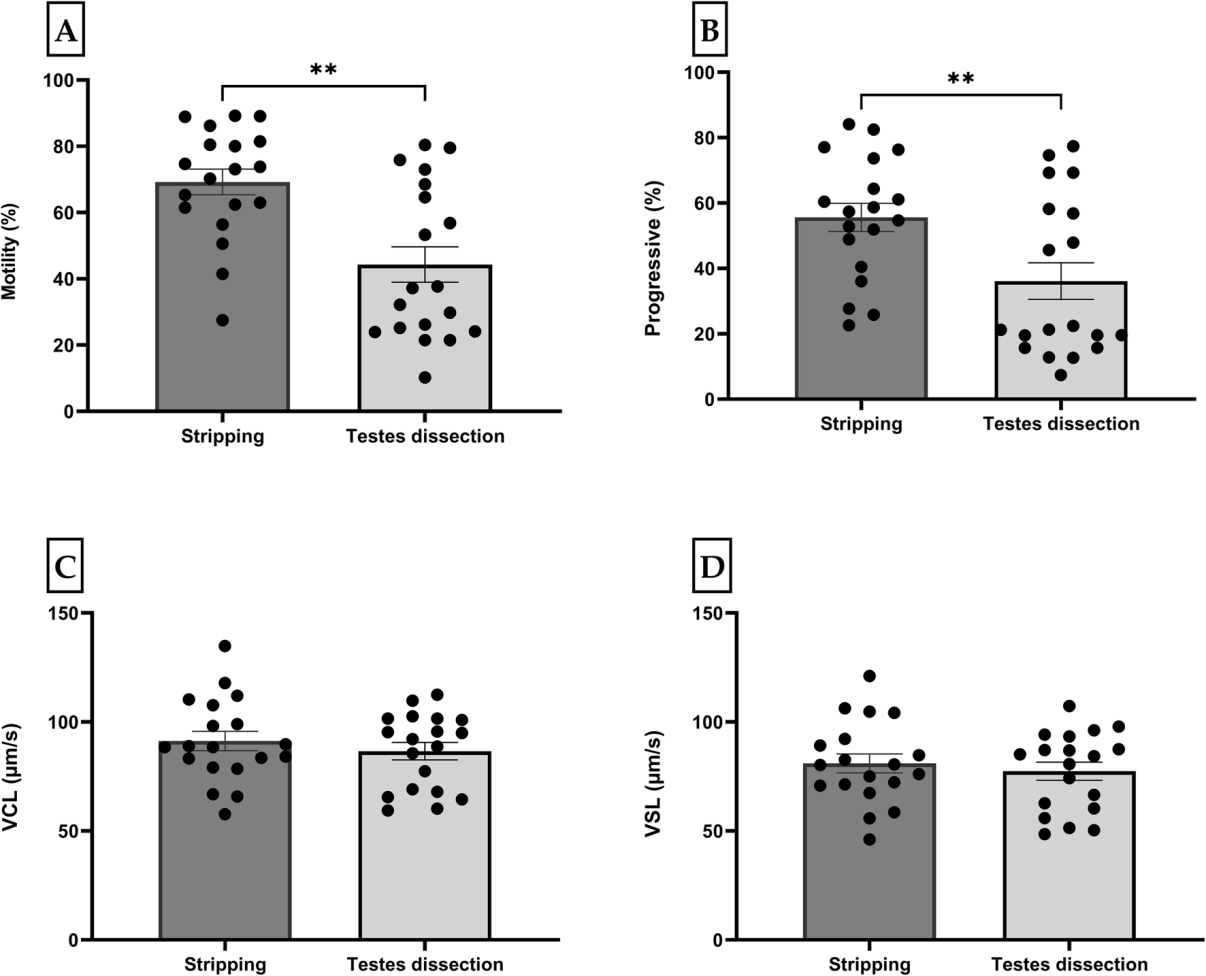
Motility parameters (Mean ± SE) of medaka spermatozoa collected by two different methods (stripping vs. testes dissection) (n = 19) and activated by HBSS 300: (**A**) motility rate (%); (**B**) progressivity (%); (**C**) curvilinear velocity (VCL) (μm/s); and (**D**) straight-line velocity (VSL) (μm/s). The figures present data sets that are significant at different levels: ** *p* < 0.01.

The method of milt collection had significant effects on some of the sperm parameters (Fig. 2). The total sperm motility (Fig. 2A) and progressivity (Fig. 2B) were significantly higher in milt samples extracted by stripping (70 ± 4%, *p* = 0.01 and 56 ± 4, *p* = 0.006), respectively compared to those extracted by testes dissection (43 ± 6 and 35 ± 7). There were no significant differences of other sperm kinematics parameters between these two collection methods (Fig. 2C,D and Table S2).

#### Testing different housing conditions

**Figure 3:**
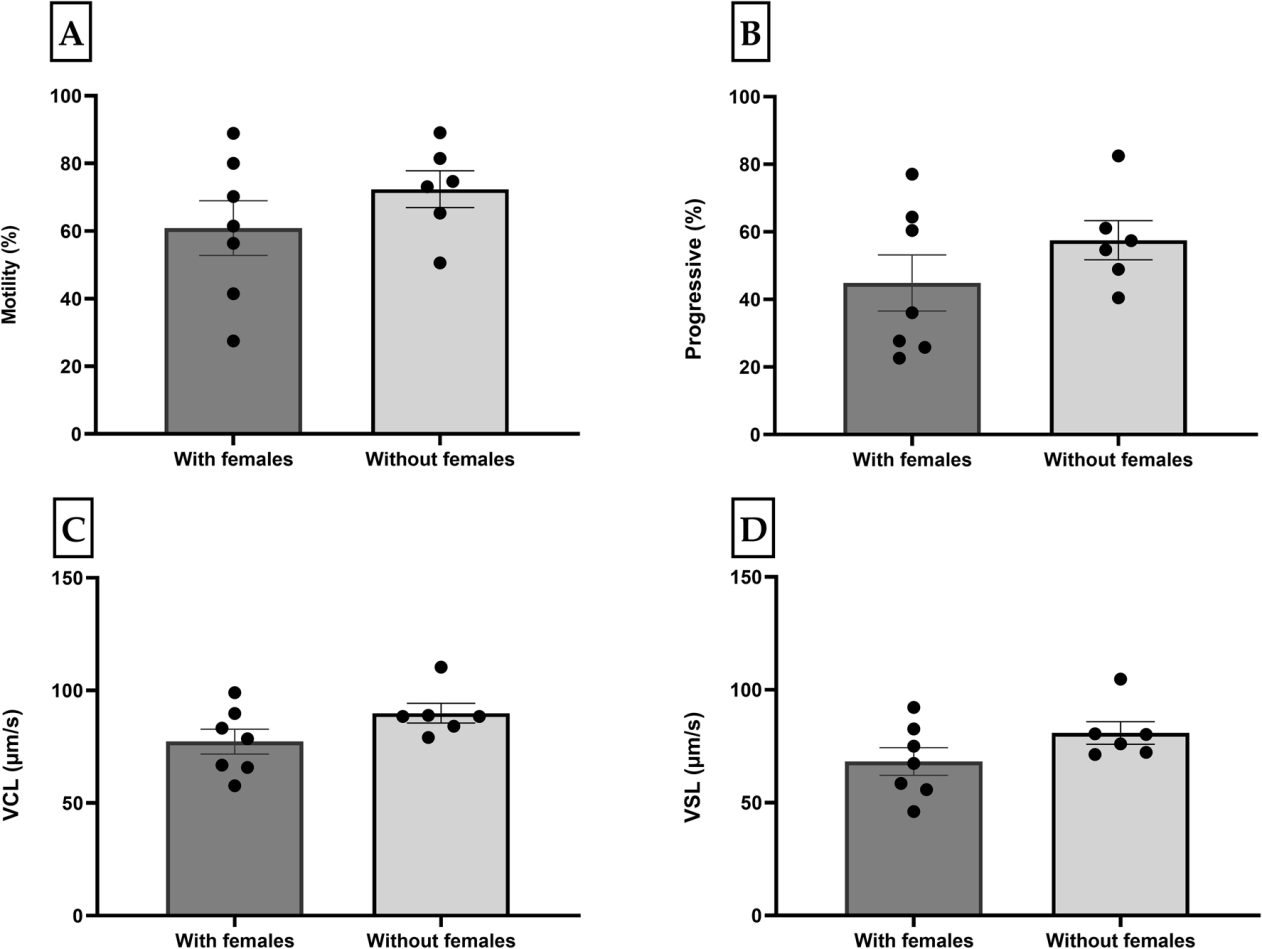
Motility parameters (Mean ± SE) of medaka spermatozoa collected by stripping from male medakas housed in two different conditions and activated by HBSS 300: (**A**) motility rate (%); (**B**) progressivity (%); (**C**) curvilinear velocity (VCL) (μm/s); and (**D**) straight-line velocity (VSL) (μm/s). With females (n = 7): the males were separated from the females one night prior to collection; Without females (n = 6): the males were kept for about one month without females prior to collection. Motility was recorded at RT.

The effect of different housing conditions on sperm quality parameters was not significant (Fig. 3). The males housed separately for a longer period had a non-significant higher total sperm motility (72 ± 5%, *p* = 0.25) (Fig. 3A), progressivity (58 ± 6%, *p* = 0.32) (Fig. 3B), VCL (90 ± 4 µm/s, *p* = 0.15) (Fig. 3C) and VSL (81 ± 5 µm/s, *p* = 0.20) (Fig. 3D) compared to the males separated only one night before collection (61 ± 8%, 45 ± 8%, 77 ± 6 µm/s and 68 ± 6 µm/s). The same effects were observed in other sperm parameters (Table S3).

### Sperm motility duration

**Figure 4:**
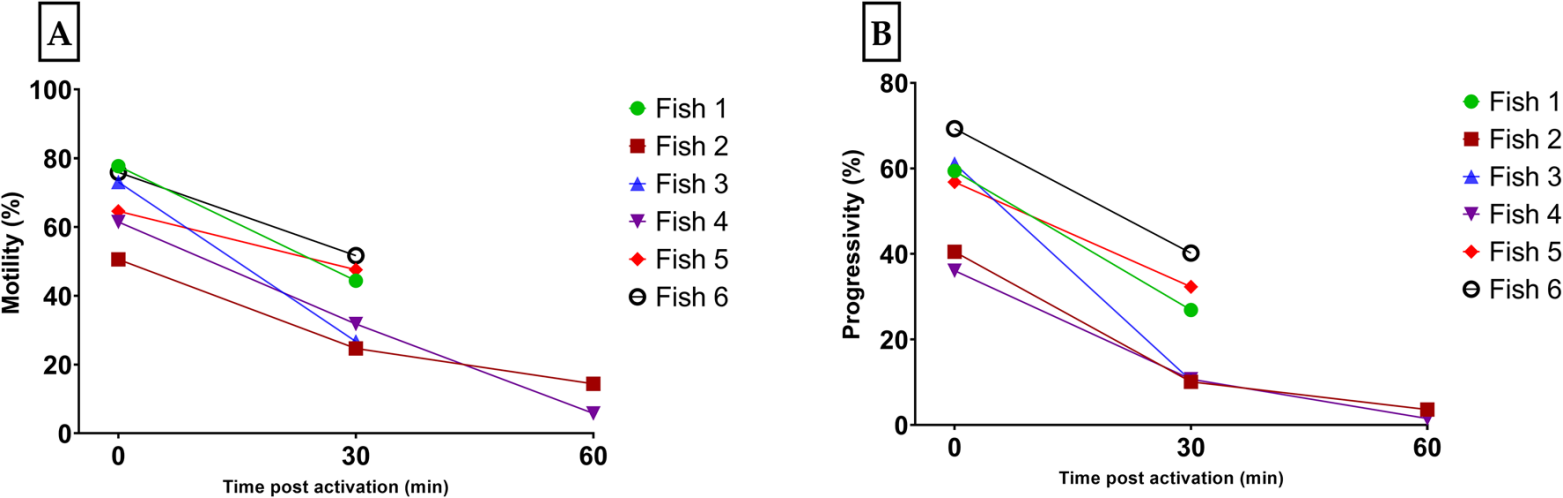
Motility parameters of stripped medaka spermatozoa at different time (0 to 60 min) post activation in HBSS 300 (0 min: 10 s): (**A**) total motility rate (%); (**B**) progressivity (%). Figures A and B show results from only two males at 60 min post activation. Activated sperm was maintained at RT for the duration of the test.

Figure 4 shows sperm movement duration after activation with HBSS 300 at RT in six individual males. The sperm was still slightly motile, although very slow, one hour after activation. Both total sperm motility (Fig. 4A) and progressivity (Fig. 4B) were reduced by only 30% after 30 min post activation. The other sperm motility parameters were also reduced (Table S4).

### Sperm storage study

#### Testing of short-term storage

During sperm storage on ice, the storage time had no significant effect on the extent of sperm immobilization. However, the percentage of immobile sperm varied significantly among the immobilization medium at different osmolalities. A significantly higher percentage of immobilization was observed in HBSS 600 (97 ± 2%) compared to HBSS 500 (93 ± 4%) after 4 h storage (*p* = 0.007).

**Figure 5:**
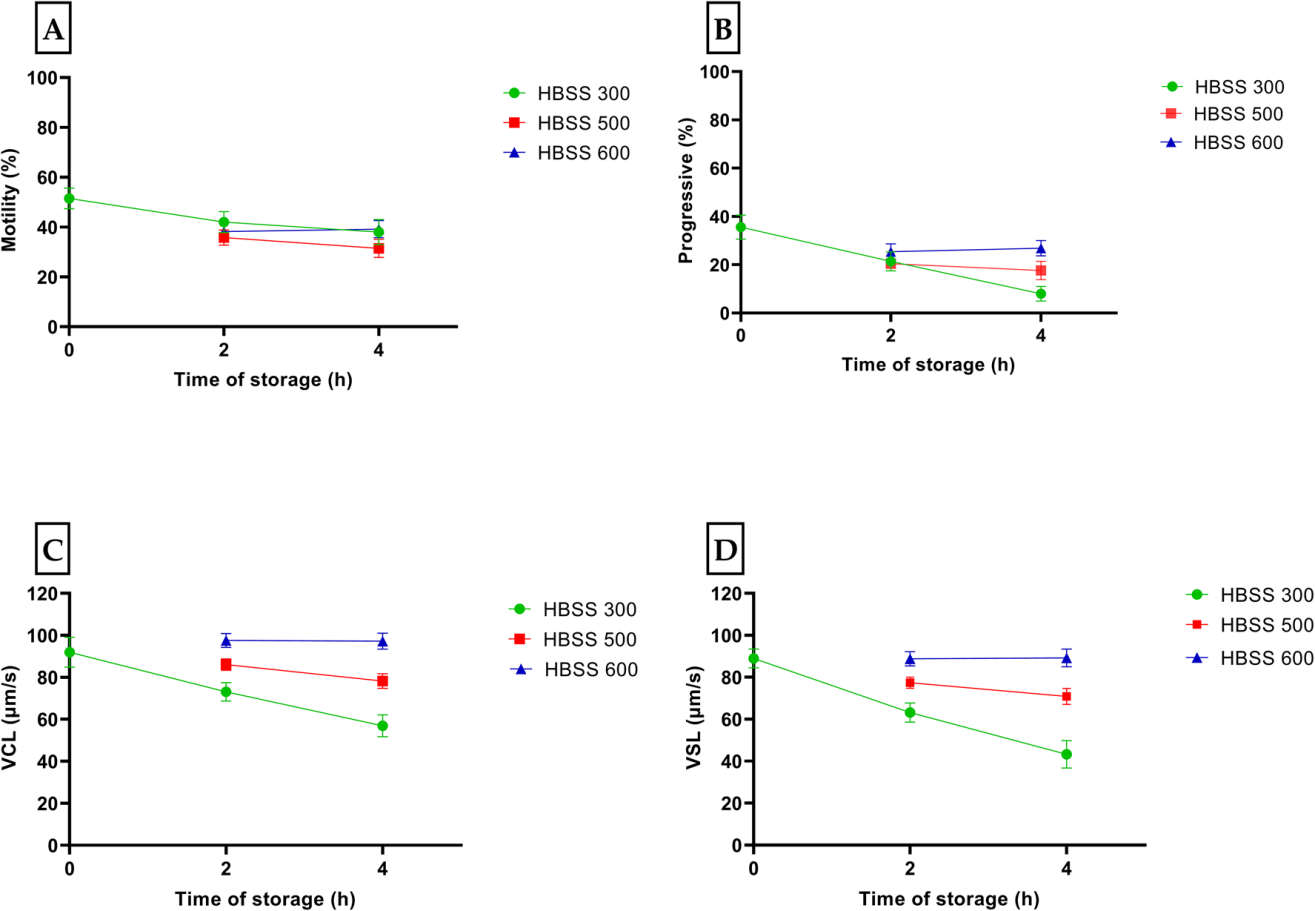
Motility parameters (Mean ± SE) of medaka spermatozoa collected from 12 males, stored on ice with different extenders over time (10 s (0 h), 2 h and 4 h), and activated by HBSS 300 at RT: (**A**) motility rate (%); (**B**) progressivity (%); (**C**) curvilinear velocity (VCL) (μm/s); and (**D**) straight-line velocity (VSL) (μm/s).

Figure 5 shows the results from activation test trial after different storage time. In this trial, the storage time on ice had no significant effect on total sperm motility (Fig. 5A) and progressivity (Fig. 5B). On the other hand, the effect of storage time on VCL (Fig. 5C) and VSL (Fig. 5D) was significant (*p* = 0.02). There was a significant reduction (13%) in progressivity of the sperm stored in HBSS 300 compared to HBSS 500 (2%, *p* = 0.04) and HBSS 600 (0%, *p* = 0.002) after 2 h storage on ice (from 2 to 4 h). The same significant effect was observed in VCL and VSL between HBSS 300 and the two other extenders.

#### Post-thaw motility of sperm cryopreserved using different immobilization solutions.

Based on the results from the short-term storage study, the osmolality of 600 mOsm/kg during storage was shown to maintain the sperm motility and progressivity performances (after activation) better than the two other osmolalities. Therefore, this 600 mOsm/kg osmolality was selected for further analysis of sperm cryopreservation, using the same cryoprotectant but different extenders (HBSS vs. Kurokura) at 600 mOsm/kg. In this long-term storage study, we tested cryosurvival of the samples immediately after thawing and after a one-hour post-thaw storage on ice. Although the results did not reach statistical significance, cryosurvival factor was slightly higher in Kurokura 600 compared to HBSS 600 (*p* = 0.2) (Table 1). Interestingly, one hour storage on ice after thawing did not significantly change the cryosurvival factor (*p* = 0.3*)*. This non-significant effect was also observed when the type of extender was excluded from the statistical model (see Supplementary Fig. S1).

**Table 1:** Impact of different immobilization solutions on sperm cryosurvival factor (CSF %) of medaka sperm immediately after thawing and after one-hour post-thaw storage on ice thawing.

Sperm parameters	HBSS 600 (n = 3)	Kurokura 600 (n = 4)
CSF: Post-thaw (0 h)	24.28 ± 3.04	30.25 ± 3.22
CSF: Post-thaw (1 h on ice)	33.75 ± 0.80	26.99 ± 7.20

No statistical differences between extenders, and between post-thaw times (Mean % ± SE).

## Discussion

The use of fish sperm parameters as an endpoint in experiments depends on the choice of efficient and reliable methods for collection, storage, and initialization of motility. In addition, in studies that aim to evaluate sperm parameters before and after cryopreservation, choosing a suitable extender or immobilization solution is as crucial as choosing a cryoprotectant. The time and storage methods, from thawing to analysis or in vitro* fertilization* (IVF) using frozen-thawed sperm, are also among the factors that can affect research outcomes.

### Sperm motility initiation

In most externally fertilizing fish species, sperm remain immotile within the testes until released into an external medium, water, in their natural habitat. The initiation of sperm motility depends on the ion composition and osmolality of the surrounding environment. For marine fishes, the suitable conditions for motility activation involve a hyperosmotic external medium compared to the seminal fluid. On the other hand, for freshwater fishes, the optimal conditions require an external medium that is hypo-osmotic relative to the seminal fluid^15^. In an experimental context, contamination of the sperm sample with urine during extraction can lead to sperm activation. Since fish sperm become motile immediately after activation and the motility decreases after a short time (10–20 s), it is necessary to avoid contamination with water and urine during the extraction and, not least, to use a suitable immobilization solution to inhibit sperm motility prior to evaluation or cryopreservation^14^.

#### Activation extenders

In previous studies examining medaka sperm, HBSS (Hanks' Balanced Salt Solution) or Yamamoto solution has been commonly used as the activating medium^8,10,11,17^. According to a specific study on medaka sperm^2^, it was observed that the sperm motility was initiated in HBSS solutions with osmolalities below 686 mOsm/kg. The authors reported the highest level of motility within the osmolality range of 25 to 227 mOsm/kg. To the best of our knowledge, there are no previous studies that compare different activation mediums with different compositions in medaka. In the current study, we compared two different activation solutions at approximately the same osmolalities (~ 300 mOsm/kg), HBSS 300 and K 180 (called K 300 in our study). We did not find significant differences in total sperm motility and progressivity. However, the sperm activated by K 300 demonstrated significantly higher values for VCL and VSL compared to those activated by HBSS 300 (Fig. 4C,D). Comparable significant differences were also observed in VAP (Table S2). One of the difficulties encountered in this study was using a single individual to test various extenders for activation. This posed a challenge due to the difficulty of dividing a milt sample while maintaining sample homogeneity in small model fish. Consequently, we used the milt from different individuals to test the effect of activation medium. While it is more aligned with natural conditions for sperm to become active in the water where the fish raises and acclimates^14^, only a limited number of previous studies have used aquarium water for medaka sperm activation. In a previous study, the sperm were immotile in aquarium water^13^. In the current study, we observed either a complete absence of motility or very short motility lasting less than a few seconds in aquarium water (data are not shown). This short-term motility posed challenges in systematically recording and analyzing samples' motility after contact with aquarium water. Notably, the osmolality of the aquarium water used in this study was approximately 10 mOsm/kg. Interestingly, natural sperm activation mode in medaka may be attributed to the interaction with the ovarian fluid. While fish sperm is typically activated in the surrounding water, the presence of ovarian fluid has been observed to enhance or extend the duration of sperm motility in zebrafish^18^ and some other teleost^19,20^. In the present study, medaka sperm were activated at an osmolality of approximately 300 mOsm/kg, which corresponds to ovarian fluid osmolality^19^. Considering the similarity in osmolality between seminal plasma and ovarian fluid, it is conceivable that the ionic composition of medaka ovarian fluid may also play a crucial role in the activation process^19,21,22^. Altogether, it can be suggested that the role of oocyte fluid is particularly significant in initiating sperm motility and for maintaining the structural and functional integrity of medaka spermatozoa. However, further investigation is necessary to elucidate the specific role of ovarian fluid and ions in the activation of medaka sperm.

#### Sperm collection methods

In most previous studies, medaka sperm was collected after dissection of the testes^2,8,9^, and to the best of our knowledge, there is only one study that used the sample collected by stripping not into the tubes but directly in a cavity slide containing test solution^10^. In our previous study, we demonstrated the technique of sperm collection by abdominal massage into capillary tubes^13^. Sperm collection using stripping under anesthesia has several advantages over testes dissection. This technique is more reliable for the evaluation of sperm parameters since the milt sample is less contaminated with red blood cells, which can affect overall sperm quality^23^ and hinder the good visualization of the spermatozoa^24^. Sperm maturation has been observed to occur in the spermatic duct in some fish species, including salmonids^25,26^. Therefore, sperm samples retrieved after testes dissection can be less mature or have more heterogeneous maturation status than the sperm collected by stripping. In addition, stripping under anesthesia makes it possible, after fish recovery from some days, to take the sample from the same fish several times during the experimental studies. This issue can be additionally relevant to 3R's principle (particularly Reduction) in the design of studies. In the present study, total sperm motility and progressivity in milt collected by stripping were significantly higher than testicular milt. These results are similar to the findings from previous studies using smelt (*Osmerus eperlanus L*.)^27^ and Eurasian perch (*Perca fluviatilis*)^28^. In contrast, it was reported a similar total motility of the sperm collected by stripping and abdominal massage in zebrafish compared to the dissection^29^ or even higher total motility and progressive motility of the sperm collected by testes dissection in sauger (*Sander canadensis*)^30^ and medaka^13^. However, in the mentioned study in sauger, a higher number of sperm with abnormal morphology was reported in testicular sperm samples^30^. An additional factor contributing to the lower sperm motility parameters in testicular sperm recorded in the present study, alongside those mentioned earlier, could be attributed to our study’s methodology wherein testicular sperm was obtained from the same fish that underwent milt collection through stripping at the short time prior to testes dissection. A portion of this decline may be linked to some practical limitations of the experimental design. To avoid significant deviations, we did not empty the entire sperm reservoir by stripping. However, to gain deeper insights in the future, it is worth to conduct additional research by collecting testicular sperm samples at longer intervals post-stripping from the same group of fish.

#### Housing conditions

We tested males separated from the females because we hypothesized that they would not naturally release sperm as often as those stimulated by the presence of the females, which may lead to aging of the sperm. Sperm ageing inside the male refers to male sperm storage and can impact sperm quality in the different ways. Sperm undergo ageing caused by oxidative stress while stored in the male body^31^. DNA damage from oxidative stress was also reported in aged zebrafish sperm^32^. Male sperm storage can affect not only the sperm quality of the males^13,32–34^ but also their offspring’s reproductive fitness^34^. We did not find significant effects in the sperm quality parameters between the males housed separately and co-housed with the females. However, some previous studies have reported that male sperm storage reduced sperm quality in guppy (*Poecilia reticulata*)^33,34^ and zebrafish^32^. In our previous study using Hd-rR strain medaka at approximately the same ages, we recorded higher sperm motility in males separated from females for one month^13^. While teleost fish share some similarities regarding their reproductive biology, there are notable differences in their reproductive physiology and strategies. Since we could find a slightly higher (non-significant) sperm quality in aged medaka sperm in two independent studies with two different strains of medaka, further experiments with larger sample sizes are strongly suggested to shed light on the effect of male sperm storage in this teleost fish. An increased sample size in future studies could potentially reveal significant effects that were not apparent in our study, thereby providing a more comprehensive understanding of the phenomena under investigation.

### Sperm motility duration

The sperm motility duration is an important parameter in fish sperm studies. This is controlled by ionic and osmotic factors in fish, which can affect sperm membrane integrity and result in a shorter period of motility after activation^21^. For practical reasons, it is important to know how long fish sperm remain motile after motility initiation. An example is when one is intended to optimize methods for sperm handling or the choice of fish models in experimental studies. Zebrafish sperm, the most used fish species in experimental studies, are motile approximately 60 s after activation^35^. In the present study, medaka sperm activated in HBSS 300 and stored at RT were still motile 30 min after activation. This is in agreement with previous studies that reported motility duration for a long period after activation of sperm stored at 4 °C^2^, 27 °C and on ice^13^. This finding suggests that medaka sperm may be deemed more suitable for experimental investigations due to their reduced susceptibility to variations in timing between motility initiation and assessment across samples and replicates.

### Sperm storage

Short-term storage of sperm after collection provides flexibility in experimental conditions. This is also important concerning the storage of sperm before fertilization in both experimental and commercial contexts. In aquaculture, short-term storage enables the effective handling of broodfish, preventing disease transmission and reducing the need for a larger fish broodstock^36^. For cryopreservation protocols, finding a proper immobilization medium for short-term storage is essential to maintain both motility and progressivity before and after freezing. The present study showed that storage of the medaka sperm in the extenders with osmolalities between 300 and 600 mOsm/kg could maintain sperm motility during 4 h storage on ice. However, storage of the sperm in an extender at 600 mOsm/kg could better maintain the sperm progressive motility and velocity. This is in accordance with the only previous study on medaka that tested the different osmolalities of the extenders, and suggested that the osmolalities ranging between 274 and 500 mOsm/kg can maintain sperm motility^2^. However, progressive motility parameters were not included in this study, while it allowed us to demonstrate a small advantage of 600 over 300 mOsm/kg. Positive correlations between progressive sperm motility and velocity parameters and fertilization rate were previously reported in African catfish (*Clarias gariepinus*)^37^ and streaked prochilod (*Prochilodus lineatus*)^38^. Therefore, based on the results from the present study, an extender at an osmolality of 600 mOsm/kg can be considered the best choice for sperm immobilization in medaka. Further investigation will be required to examine the motility and progressivity parameters of the medaka sperm immobilized for a longer period.

Small model fish such as zebrafish and medaka have unique characteristics that make them valuable for studying various biological processes in different research fields. Researchers can maintain a stable and accessible genetic resource for future experiments by using sperm cryopreservation. This technique ensures that the genetic diversity of these small model fish is preserved, allowing for continued scientific advancements^39^. Cryopreservation of zebrafish sperm is a well-studied area, but there is limited research on cryopreservation of medaka sperm collected by testes dissection^9^. This study evaluates the cryopreservation of medaka sperm collected by stripping using an extender for cryopreservation, with two different salines (HBSS and Kurokura). The results showed no differences in cryosurvival rate of the sperm immobilized in HBSS and Kurokura at an osmolality of 600 mOsm/kg immediately after thawing. Post-thawing storage time and conditions can affect sperm quality and fertilization rate^40^. Immediate fertilization after thawing is generally recommended for using cryopreserved fish sperm^41^. However, there is no consensus on post-thawing storage period and temperature, as it often depends on the species and the cryoprotectant used. After cryopreserving salmonid sperm in methanol, storing it at 20 °C for 1 h post-thaw did not impact fertilization rate^40^. Another study by the same research group found that the maximum fertilization capacity of cryopreserved African catfish sperm was reached 24–26 h after thawing and storage at 4 °C^42^. Our study demonstrated that storing cryopreserved sperm on ice after thawing maintained medaka sperm motility parameters, regardless of the type of immobilization medium used. However, there was a slight advantage observed with HBSS 600 as the extender in cryopreservation solution. These results suggest that short-term storage of post-thawed sperm on ice could be a practical option when immediate fertilization is not possible after thawing. Further investigations involving different temperatures, sperm evaluation, and fertilization at various time points post-thawing can help in developing a more reliable protocol for post-thawing sperm handling in medaka.

## Conclusion

This current study allowed us to select an efficient method for sperm collection, storage, and motility activation in medaka. We demonstrated that choosing suitable extenders and appropriate timing and storage methods could improve sperm quality. The study also sheds light on the benefits of collecting milt by stripping rather than from testicular tissue. Additionally, housing conditions of males, either co-housing with females or with males only, had little influence on sperm quality. The investigation of cryopreservation methods using different extenders provides information about the extender's type and osmolality that can contribute to the advancement of cryopreservation techniques in medaka sperm. The study suggests that storage (1 h) of post-thawed medaka sperm on ice is a practical option when immediate fertilization is not feasible, demonstrating its potential for extending the usability of cryopreserved sperm. While the insights gained from this study provide guidance in selecting suitable methods and conditions to enhance the accuracy and reliability of fish sperm-related studies, future research should focus on assessing the fertilization rates and potential effects of different storage and cryopreservation methods on offspring development and reproductive success at both morphological, functional, and molecular levels.

### Supplementary Information

Supplementary Information 1.

Supplementary Video 1.

## Data Availability

The datasets used or analyzed during the current study are available from the corresponding author on reasonable request.

## References

[1] Kinoshita M, Murata K, Naruse K, Tanaka M (2009). Medaka: Biology, Management, and Experimental Protocols. Medaka.

[2] Yang H, Tiersch TR (2009). Sperm motility initiation and duration in a euryhaline fish, medaka (*Oryzias latipes*). Theriogenology.

[3] Wan T (2022). Assessment of parental benzo[a]pyrene exposure-induced cross-generational neurotoxicity and changes in offspring sperm DNA methylome in medaka fish. Environ. Epigenetics.

[4] Li F (2020). Identification, expression and functional analysis of prmt7 in medaka *Oryzias latipes*. J. Exp. Zool. B Mol. Dev. Evol..

[5] Kondo Y, Kohda M, Koya Y, Awata S (2020). Sperm allocation strategies depending on female quality in medaka (*Oryzias latipes*). Zool. Sci..

[6] Closs LE (2023). Artificial light at night disrupts male dominance relationships and reproductive success in a model fish species. Sci. Total Environ..

[7] Yan S, Liang M, Chen R, Hong X, Zha J (2020). Reproductive toxicity and estrogen activity in Japanese medaka (*Oryzias latipes*) exposed to environmentally relevant concentrations of octocrylene. Environ. Pollut..

[8] Kowalska A, Kamaszewski M, Czarnowska-Kujawska M, Podlasz P, Kowalski RK (2020). Dietary ARA improves COX activity in broodstock and offspring survival fitness of a model organism (Medaka *Oryzias latipes*). Animals.

[9] Yang H, Norris M, Winn R, Tiersch TR (2010). Evaluation of cryoprotectant and cooling rate for sperm cryopreservation in the euryhaline fish medaka *Oryzias latipes*. Cryobiology.

[10] Hara Y, Strüssmann CA, Hashimoto S (2007). Assessment of short-term exposure to nonylphenol in Japanese medaka using sperm velocity and frequency of motile sperm. Arch. Environ. Contam. Toxicol..

[11] Hashimoto S (2009). Effects of ethinylestradiol on medaka (*Oryzias latipes*) as measured by sperm motility and fertilization success. Arch. Environ. Contam. Toxicol..

[12] Kawana R, Strüssmann CA, Hashimoto S (2003). Effect of p-Nonylphenol on sperm motility in Japanese medaka (*Oryzias latipes*). Fish Physiol. Biochem..

[13] Closs L, Sayyari A, Fontaine R (2022). Sperm collection and computer-assisted sperm analysis in the teleost model Japanese medaka (*Oryzias latipes*). J. Vis. Exp..

[14] Beirão J (2019). Sperm handling in aquatic animals for artificial reproduction. Theriogenology.

[15] Alavi SMH, Cosson J (2006). Sperm motility in fishes (II). Effects of ions and osmolality: A review. Cell Biol. Int..

[16] Depincé A (2020). DNA methylation stability in fish spermatozoa upon external constraint: Impact of fish hormonal stimulation and sperm cryopreservation. Mol. Reprod. Dev..

[17] Kowalska A, Siwicki AK, Kowalski RK (2017). Dietary resveratrol improves immunity but reduces reproduction of broodstock medaka *Oryzias latipes* (Temminck & Schlegel). Fish Physiol. Biochem..

[18] Poli F, Immler S, Gasparini C (2019). Effects of ovarian fluid on sperm traits and its implications for cryptic female choice in zebrafish. Behav. Ecol..

[19] Zadmajid V, Myers JN, Sørensen SR, Ernest Butts IA (2019). Ovarian fluid and its impacts on spermatozoa performance in fish: A review. Theriogenology.

[20] Rusco G (2023). The use of ovarian fluid as natural fertilization medium for cryopreserved semen in mediterranean brown trout: The effects on sperm swimming performance. Vet. Sci..

[21] Cosson J (2004). The ionic and osmotic factors controlling motility of fish spermatozoa. Aquacult. Int..

[22] Wang X, Liu Q, Zhou L, Song Z, Li J (2023). Effect of ovarian fluid on sperm performance in teleost with internal and external fertilization strategies. Theriogenology.

[23] Henkel R (2005). Effect of reactive oxygen species produced by spermatozoa and leukocytes on sperm functions in non-leukocytospermic patients. Fertil. Steril..

[24] Verheyen G, Popovic-Todorovic B, Tournaye H (2017). Processing and selection of surgically-retrieved sperm for ICSI: A review. Basic Clin. Androl..

[25] Kowalski RK (2012). Quality and quantity of smelt (*Osmerus eperlanus* L.) sperm in relation to time after hormonal stimulation. Reprod. Biol..

[26] Schulz RW (2010). Spermatogenesis in fish. Gen. Comp. Endocrinol..

[27] Kowalski RK (2006). Semen biology and stimulation of milt production in the European smelt (*Osmerus eperlanus* L.). Aquaculture.

[28] Alavi SH (2008). Physiology and behavior of stripped and testicular sperm in *Perca fluviatilis* L. 1758. Cybium.

[29] Jing R, Huang C, Bai C, Tanguay R, Dong Q (2009). Optimization of activation, collection, dilution, and storage methods for zebrafish sperm. Aquaculture.

[30] Blawut B (2020). Testicular collections as a technique to increase milt availability in *sauger* (*sander canadensis*). Anim. Reprod. Sci..

[31] Reinhardt K (2007). Evolutionary consequences of sperm cell aging. Q. Rev. Biol..

[32] Cattelan S, Gasparini C (2021). Male sperm storage impairs sperm quality in the zebrafish. Sci. Rep..

[33] Gasparini C, Kelley JL, Evans JP (2014). Male sperm storage compromises sperm motility in guppies. Biol. Lett..

[34] Gasparini C, Dosselli R, Evans JP (2017). Sperm storage by males causes changes in sperm phenotype and influences the reproductive fitness of males and their sons. Evolut. Lett..

[35] Wilson-Leedy JG, Kanuga MK, Ingermann RL (2009). Influence of osmolality and ions on the activation and characteristics of zebrafish sperm motility. Theriogenology.

[36] DeGraaf JD, Berlinsky DL (2004). Cryogenic and refrigerated storage of Atlantic cod (*Gadus morhua*) and haddock (*Melanogrammus aeglefinus*) spermatozoa. Aquaculture.

[37] Rurangwa E, Volckaert FAM, Huyskens G, Kime DE, Ollevier F (2001). Quality control of refrigerated and cryopreserved semen using computer-assisted sperm analysis (CASA), viable staining and standardized fertilization in African catfish (*Clarias gariepinus*). Theriogenology.

[38] Viveiros ATM, Nascimento AF, Orfão LH, Isaú ZA (2010). Motility and fertility of the subtropical freshwater fish streaked prochilod (*Prochilodus lineatus*) sperm cryopreserved in powdered coconut water. Theriogenology.

[39] Yang H, Tiersch TR (2009). Current status of sperm cryopreservation in biomedical research fish models: Zebrafish, medaka, and Xiphophorus. Comp. Biochem. Physiol. Part C: Toxicol. Pharmacol..

[40] Horváth Á (2015). Post-thaw storage of sperm from various salmonid species. J. Appl. Ichthyol..

[41] Kopeika E, Kopeika J, Zhang T, Day JG, Stacey GN (2007). Cryopreservation of Fish Sperm. Cryopreservation and Freeze-Drying Protocols.

[42] Kovács É (2010). Quality of cryopreserved African catfish sperm following post-thaw storage. J. Appl. Ichthyol..

