# Supplementary material for: Sperm handling and management in the teleost model fish Japanese medaka (Oryzias latipes)
Source: Sci Rep. 2024 Jun 26;14:14736. doi: 10.1038/s41598-024-65376-8 (PMC11208518; doi:10.1038/s41598-024-65376-8)
Supplement: Supplementary file 1 — Supplementary Information 1. [file 41598_2024_65376_MOESM1_ESM.docx]

**Supplementary information**

**Sperm handling and management in the teleost model fish Japanese Medaka (Oryzias latipes)**

Amin Sayyari ^*^, Anette Kristine Krogenæs, Ian Mayer and Catherine Labbé

[amin.sayyari@nmbu.no](mailto:amin.sayyari@nmbu.no)*

* Corresponding authors


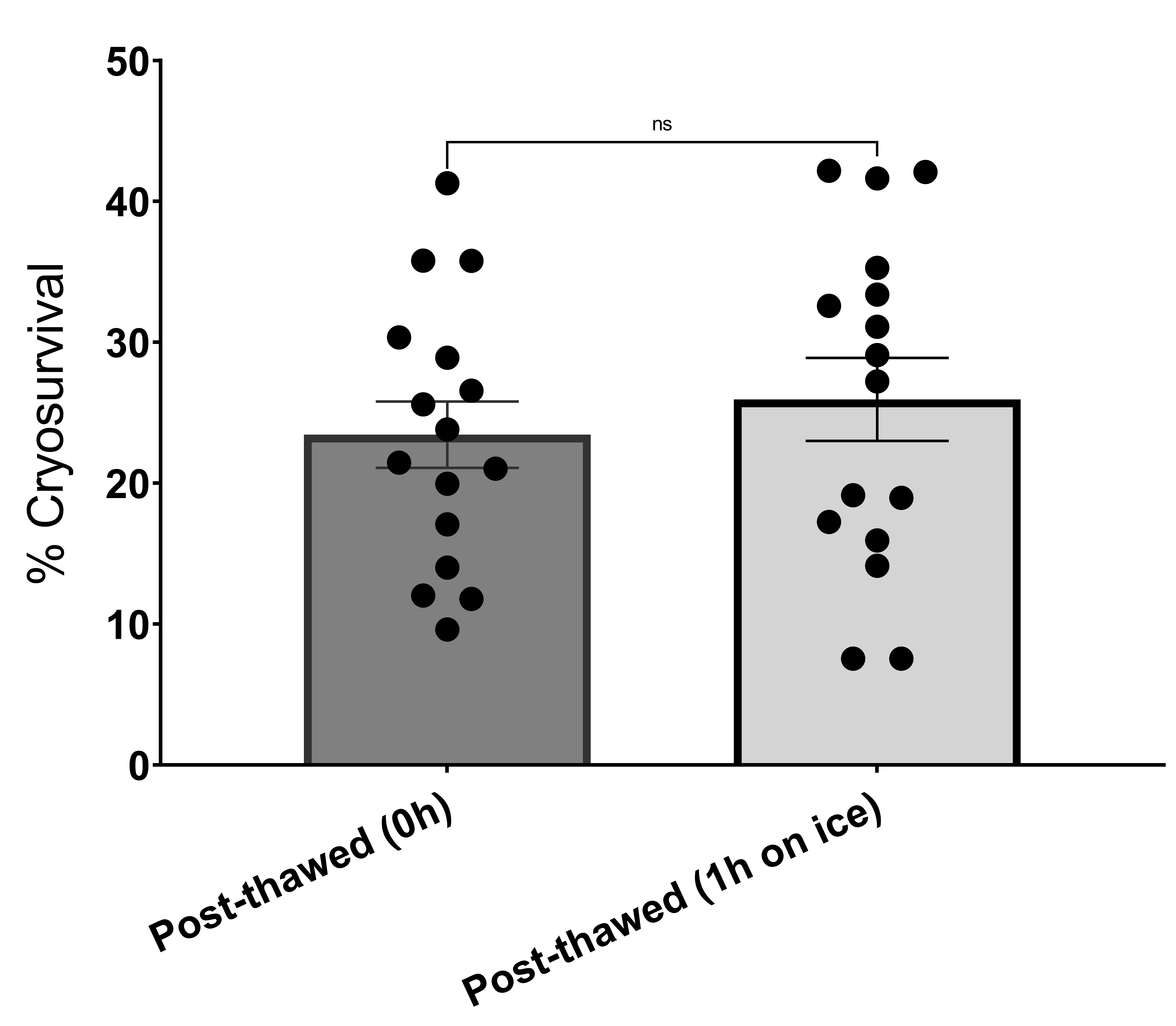


**Fig. S1** Cryosurvival (Mean % ± SE) of medaka sperm immediately after thawing (0 h) and 1 hour storage on ice after thawing (1 h on ice) regardless type of immobilization medium.

|  | **Activation solution** | | ***p* value** |
| --- | --- | --- | --- |
|  | **HBSS 300 (n=13)** | **K 180 (n=6)** |  |
| LIN % | 85 ± 2 | 86 ± 2 | 0.61 |
| STR % | 96 ± 1 | 95 ± 1 | 0.68 |
| VAP (µm/s) | 76 ± 4 | 99 ± 7 | 0.02 |
| WOB % | 88 ± 1 | 90 ± 1 | 0.38 |

**Table S1.** Motility parameters (Mean ± SE) of medaka spermatozoa immediately after milt collection activated by HBSS (n=13) and Kurokura (n=6).

|  | **Sperm collection method** | | ***p* value** |
| --- | --- | --- | --- |
|  | **Stripping (n=19)** | **Testes dissection**  **(n = 19)** |  |
| LIN % | 85 ± 1 | 88 ± 1 | 0.12 |
| STR % | 96 ± 1 | 97 ± 1 | 0.10 |
| VAP (µm/s) | 84 ± 4 | 79 ± 4 | 0.68 |
| WOB % | 89 ±1 | 90 ± 1 | 0.26 |

**Table S2.** Motility parameters (Mean ± SE) of medaka spermatozoa collected by two different methods (stripping vs. testes dissection) (n=19)

|  | **Housing condition** | | ***p* value** |
| --- | --- | --- | --- |
|  | **With females (n=7)** | **Without females (n=6)** |  |
| LIN % | 84 ± 3 | 86 ± 2 | 0.38 |
| STR % | 95 ± 1 | 96 ± 1 | 0.73 |
| VAP (µm/s) | 70 ± 6 | 83 ± 5 | 0.12 |
| WOB % | 87 ± 2 | 90 ± 1 | 0.25 |

**Table S3.** Motility parameters (Mean ± SE) of medaka spermatozoa collected from male medakas housed in two different housing conditions (n=13)

|  | **Time after activation** | | ***p* value*** |
| --- | --- | --- | --- |
|  | **0 min (n=6)** | **30 min (n=6)** |  |
| LIN % | 86 ± 2 | 77 ± 3 | 0.04 |
| STR % | 96 ± 1 | 94 ± 1 | 0.08 |
| VAP (µm/s) | 81 ± 5 | 53 ± 6 | 0.01 |
| VCL (µm/s) | 87 ± 5 | 61 ± 6 | 0.01 |
| VSL (µm/s) | 79 ± 5 | 51 ± 6 | 0.01 |
| WOB % | 90 ± 2 | 82 ± 2 | 0.04 |

**Table S4.** Motility parameters of medaka spermatozoa collected from 6 males at different post activation time (10 s (0 min) and 30 min). * Effect of time on sperm motility parameters.

**Video S1.** Visualization of sperm motility recorded and analyzed by CASA: Motile sperm are identified by color-coded tracks, and static sperm are identified by red dots.
